# Transcriptomic Profiling in Low-Risk Thyroid Cancer Induced by Microwave Ablation

**DOI:** 10.1155/2024/6674506

**Published:** 2024-05-15

**Authors:** Huarong Li, Lei Liang, Jianming Li

**Affiliations:** ^1^Department of Ultrasound, Aerospace Center Hospital, Beijing 100049, China; ^2^Department of Interventional Ultrasound, Fifth Medical Center of Chinese PLA General Hospital, Beijing, China; ^3^Department of Ultrasound, Beijing Friendship Hospital, Capital Medical University, Beijing, China

## Abstract

**Background:**

Peripheral blood mononuclear cells (PBMCs) serve as the immune system's primary transportation hub outside of the affected ablated tissue. This study aims to explore the transcriptomic profiling of the immune response in PBMCs induced by microwave ablation (MWA) in low-risk thyroid cancer.

**Methods:**

For eight patients diagnosed with low-risk thyroid cancer, 10 ml of peripheral venous blood was collected before MWA as well as one day and one month after MWA. mRNA was extracted from PBMCs for transcriptome next-generation gene sequencing and qRT-PCR analyses. The plasma samples were used for chemokine detection purposes.

**Results:**

One day and one month after MWA, there were significant changes in GSEA, particularly in the NF-kappa B-TNF*α* pathway, inflammatory response, and early and late estrogen response. Common changes in differently expressed genes resulted in a significant downregulation of tumor-promoting genes (*BCL3*, *NR6A1*, and *PFKFB3*). One day after low-risk thyroid cancer MWA, GO enrichment analysis mainly revealed processes related to oxygen transport and other pathways. One month after MWA, GO enrichment analysis mainly revealed regulation of toll-like receptor signaling and other pathways. Furthermore, inflammation-related cytokines and regulatory genes, as well as tumor-promoting cytokines and regulatory genes, were downregulated after MWA.

**Conclusions:**

This study presents a comprehensive profile of the systemic immune response induced by thermal ablation for treating low-risk thyroid cancer. More significantly, this study provides valuable insight into potential references for systemic antitumor immunity of ablation against low-risk thyroid cancer. This trial is registered with ChiCTR1900024544.

## 1. Introduction

In recent decades, the prevalence of thyroid ultrasound screening and advancements in fine needle aspiration biopsy (FNAB) have contributed to a notable increase in the incidence of low-risk thyroid cancer. Low-risk thyroid cancer is defined as T1aN0M0 papillary thyroid microcarcinoma (PTMC) with the following characteristics: solitary lesion, diameter less than 1 cm, and nonmetastatic lymph nodes. Thyroidectomy used to be the primary treatment for PTMC. However, thermal ablation, with its minimally invasive nature and good tolerance, has achieved satisfactory results comparable with surgery [1–4].

Previous studies have reported activated immune responses following thermal ablation [5]. However, the antitumor immune response resulting from thermal ablation is predominantly studied through histological analysis. Remarkably, peripheral blood serves as the immune system's primary transportation hub beyond the ablated tissue [6, 7]. Peripheral blood mononuclear cells (PBMCs) are the peripheral immune cells, including lymphocytes, monocytes, and dendritic cells, responsible for initiating the systemic immune response directed against the targeted ablated organs [8]. Cytotoxic T lymphocytes (CTLs), as the primary effector cells for antitumor activity, can migrate and infiltrate the tumor microenvironment, where they can identify antigens and target tumor cells via the MHC class I pathway. Once the tumor cells are killed, the tumor antigens may be released, thus initiating a tumor immune circulation [9].

However, the immune-related transcriptome expression profile in peripheral blood for thyroid cancer has not yet been reported. To explore the changes in systemic immunity after thermal ablation, we analyzed the messenger RNA (mRNA) expression and transcriptomic profiling of PBMCs in low-risk thyroid cancer patients before and after thermal ablation.

## 2. Materials and Methods

### 2.1. Patients

This study was performed in compliance with the Declaration of Helsinki. This study was approved by the local Institutional Review Board of Beijing Friendship Hospital affiliated with Capital Medical University and Aerospace Center Hospital and written informed consent was obtained from all participating patients. The study was registered at the International Clinical Trial Registration (ChiCTR1900024544). From June to July 2020, eight patients diagnosed with PTMC were enrolled from Beijing Friendship Hospital affiliated with Capital Medical University and Aerospace Center Hospital, and peripheral venous blood (10 ml) was collected before thermal ablation, as well as one day and one month after thermal ablation according to the recommendations of the thyroid ablation guideline [10] and the systemic immune changes of previous studies reported [11, 12]. The extracted mRNA from PBMCs was used for RNA sequencing (RNA-seq) analysis.

### 2.2. Inclusion/Exclusion Criteria

All participating patients met the following inclusion criteria: (1) confirmation of thyroid cancer via pathology, (2) presence of a single lesion, (3) maximum lesion diameter ≤10 mm, (4) no thyroid capsule invasion, (5) no trachea and esophagus invasion, and (6) no lymph node metastases or distant metastases by imaging examination (such as ultrasound and CT). Exclusion criteria included the following: (1) pathology-confirmed presence of other types of thyroid malignancy, such as medullary carcinoma, (2) presence of cervical lymph node metastasis or distant metastasis, (3) pregnancy, (4) severe heart, respiratory, liver, or renal failure, and (5) coagulopathy with a severe bleeding tendency.

All patients were prohibited from taking antiplatelet or anticoagulant drugs at least one week before thermal ablation.

### 2.3. Thermal Ablation

Ablation schemes were tailored to the size and location of thyroid tumor and its relation to surrounding organs. A cooled-shaft microwave ablation (MWA) system (KY-2000, Kangyou Medical, China) was used in the procedure. Each application of microwave energy (30 W, 2450 MHz) lasted 20–120 s in the tumor until complete coagulative necrosis.

### 2.4. PBMC Isolation

PBMCs were isolated from 10 ml of blood via Ficoll-Hypaque gradient centrifugation. 10 ml of fresh peripheral blood was collected using an EDTA anticoagulant tube and centrifuged at 500 g for 4 min, and then, plasma was collected. An equal volume of Dulbecco's phosphate-buffered saline (DPBS) was added to the cell composition, blown, and mixed with a pipette gun. Then, the human lymphocyte separation solution was added, and the cell composition was slowly spread on the surface of the human lymphocyte separation solution. The cell solution (cell composition and human lymphocyte separation solution) was centrifuged at the rate of 800 g for 20 min, the speed was increased by 4 g/s, and the speed was decreased by 2 g/s. After centrifugation, the middle layer (PBMC composition) is drawn. Equal volume MACS buffer solution (2.5 g fetal bovine serum + 500 ml DPBS + 2 ml 0.5 M EDTA) was added, mixed upside down, centrifuged at 500 g for 4 min, and left white precipitate. Finally, it was washed three times with phosphate buffer solution (PBS). Once the third washing was completed, PBMCs were isolated and added to 1 ml of TRIzol and stored at −80°C.

### 2.5. mRNA Extraction

Total mRNA from PBMCs was extracted using TRI reagent or an Eastep Super Total RNA Extraction Kit (LS1040, Shanghai Promega) in accordance with the manufacturer's instructions. The integrity of the mRNA was verified using the Agilent Technologies 2100 bioanalyzer (Agilent Technologies, USA), and samples with an mRNA integrity number (RIN) ≥ 7 were selected for further analysis. The content and purity of total mRNA were determined using an ultraviolet photometer. Subsequently, mRNA samples were stored at −80°C and utilized for RNA-seq and qRT-PCR analyses.

### 2.6. Bulk RNA-Seq Analysis

Bulk RNA-seq analysis was executed following previously described methods [13]. Genedenovo Biotechnology Co., Ltd. (Guangzhou, China) conducted library construction and RNA-seq. The obtained RNA-seq data were normalized using fragment per kilobase of transcript per million mapped reads (FPKM) through StringTie. DESeq2 software was utilized to analyze differential expression genes (DEGs) between two groups, whereby transcripts displaying differential expression (DE) with a fold change ≥2 and *p* < 0.05 were identified by comparison.

### 2.7. GO and KEGG Pathway Analyses

Gene ontology (GO) annotation and Kyoto Encyclopedia of Genes and Genomes (KEGG) pathway analyses were carried out to elucidate the functions of all DEGs identified. GO analysis was employed to annotate the attributes and gene products and to investigate the enrichment of biological processes in DE mRNAs (https://www.geneontology.org). Pathway analysis was conducted using the KEGG analysis tool (https://www.genome.jp/kegg/) to identify the enriched biological pathways in the DEGs.

### 2.8. Gene Enrichment Analysis (GSEA)

We analyzed a list of all gene expressions between two groups as input data for our study. To identify signaling pathways, GSEA utilized the prognosis index with the Clusterprofiler package.

### 2.9. qRT‐PCR Validation

Total mRNA was reverse-transcribed into cDNA using a PrimeScript RT Reagent Kit (RR037A, TaKaRa). The mRNA expression levels of the target genes were quantified by qRT-PCR using an ABI 7500 Sequence Detection System (Applied Biosystems). The data were normalized to *GAPDH* gene expression and quantified using the 2^−ΔΔCt^ method. The *β*-actin was used as an internal control to normalize the sample differences. The primer sequences of the target genes are shown in Table 1.

### 2.10. Detection of Cytokines and Chemokines

The plasma samples were analyzed using the Bio-Plex MAGPIX system (Bio-Rad, USA) with a floating microsphere chip platform. The samples were collected at 10000 turns/minute and centrifuged for 10 minutes to obtain the supernatant. After diluting the sample four times with the sample diluent, 100 *μ*l of the diluted sample was used for detection. The standard sample was subjected to multiple dilutions. Bio-Plex Pro Human Cytokines were utilized on 48-factor plates for sifting and detection of the plasma samples. The incubation time for the samples and antibodies was 30 minutes, and the overall incubation time was 10 minutes.

### 2.11. Statistical Analyses

Student's *t*-tests or Mann–Whitney *U* tests were employed to compare continuous variables in the different groups. Data were analyzed with SPSS 20.0 and R software version 4.1.0 (https://www.r-project.org). A *p* value ≤ 0.05 (2-sided) indicated statistical significance.

## 3. Results

### 3.1. Patients Characteristics

This study enrolled eight patients with PTMC treated by MWA. The mean age was 37.25 ± 2.49 years. The patients' characteristics are shown in Table 2.

### 3.2. Differential Genetic Expression

Compared with pre-MWA treatment (control group), a total of 277 genes showed significant differences at one day after MWA. A total of 235 genes showed significant differences at one month after MWA. In accordance with the study requirements, all differentially expressed genes with consistent alterations before and after treatment were selected. From the one-day treatment group, 91 differential genes (27 genes upregulated and 64 genes downregulated) were identified. From the one-month treatment group, 37 differentially expressed genes (6 genes upregulated and 31 genes downregulated) were selected (Table 3).

### 3.3. Analysis of Common DEGs One Day and One Month after MWA

**Figure 1:**
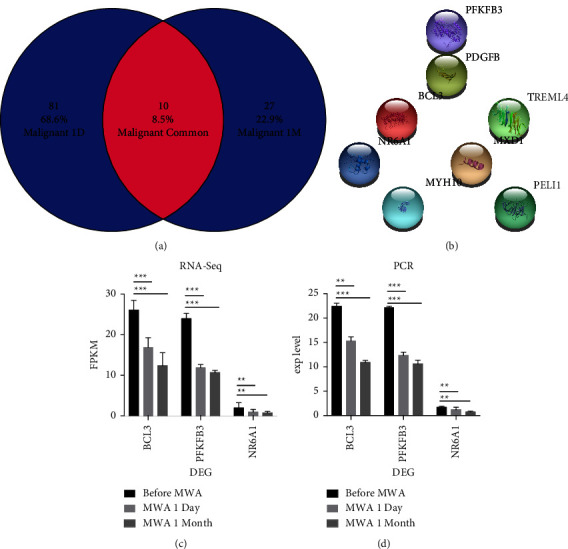
Analysis of common differential genes at 1 day (1 D) and 1 month (1 M) after MWA. A total of 10 differential genes with the same change at 1 day (1 D) and 1 month (1 M) after MWA (a) and the DEGs including *PFKFB3*, *PDGFB*, *BCL3*, and *NR6A1* (b). Protumor genes were significantly downregulated, confirmed by RNA-seq (c) and qRT-PCR (d). The data are depicted as the mean ± SD, *n* = 8 in each group. ^*∗*^*p* ≤ 0.05, ^*∗∗*^*p* ≤ 0.01; ^*∗∗∗*^*p* ≤ 0.001.

Compared with pre-MWA treatment (control group), the commonly observed DEGs (Figure 1(a)), including *AC118658.1*, *BCL3*, *CELF2-AS1*, *MXD1*, *Myh10*, *NR6A1*, *PDGFB*, *PELI1*, *PFKFB3*, and *TREML4*, exhibited changes at one day and one month after MWA (Figure 1(b)). Except for *PDGFB*, all nine DEGs were downregulated after treatment. After MWA, tumor-promoting genes such as *BCL3*, *Myh10*, *NR6A1*, and *PFKFB3* displayed downregulation (Figure 1(c)). *PELI1* was also downregulated and associated with T cell-negative regulation (Table 4). The downregulation of both tumor-promoting and T cell-negative regulatory genes after MWA indicates T cell activation and an antitumor immune response. Consistent with the RNA-seq data, significant differential expressions for selected *BCL3*, N*R6A1*, and *PFKFB3* were detected through qRT-PCR (Figure 1).

### 3.4. DEG Analysis One Day after MWA

**Figure 2:**
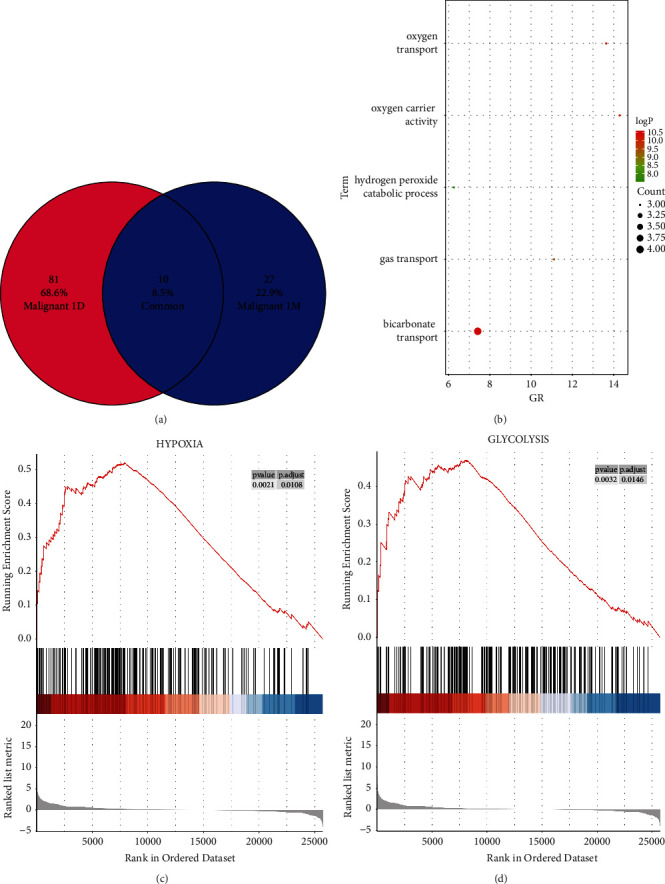
Differential gene analysis before and 1 day (1 D) after MWA. 81 differential genes (a) enriched in oxygen transport-related signaling pathways (b), consistent with GO analysis. Gene set enrichment analysis (GSEA) showed hypoxia (c) and glycolysis-related (d) signaling pathways consistent with gene ontology (GO) analysis.

After one day of MWA, GO enrichment analysis revealed that 81 genes exhibited differential expression (Figure 2(a)), with enriched functional pathways being oxygen transport, oxygen carrier activity, hydrogen peroxide catabolism, gas transport, and hydrogen carbonate transport (Figure 2(b)). The significant DEGs included *HBA1*, *HBA2*, *HBB*, and *SLC4A1*, indicating an increase in oxygen transport and relief of immune cell hypoxia after MWA (Figures 2(c) and 2(d)).

### 3.5. DEG Analysis One Month after MWA

**Figure 3:**
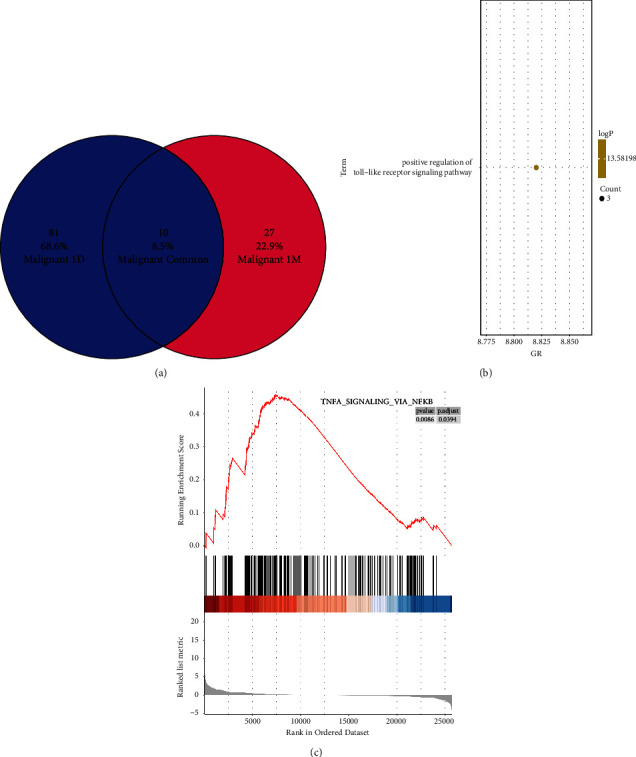
Differential gene analysis before and 1 month (1 M) after MWA. 27 differential genes (a) enriched in toll-like receptor-related signaling pathways (b). GSEA showed NF-kappa B-TNF*α* signaling pathway consistent with GO analysis (c). GSEA: gene set enrichment analysis; GO: gene ontology; MWA: microwave ablation.

After one month of MWA, a total of 27 genes demonstrated changes. GO enrichment analysis revealed that the enriched pathway was primarily related to the positive regulation of *TLR1*, *TLR2*, and *TREML4* (Figure 3(a)). These downregulated genes were found to regulate the toll-like receptor signaling pathway negatively (Figure 3(b)). The results suggest that MWA treatment for low-risk thyroid cancer may activate T cells and lead to the downregulation of tumor genes. In addition, oxygen transport relieves hypoxia, while NF-KB mediates the downregulation of the TNF pathway (Figure 3(c)).

### 3.6. GSEA

**Figure 4:**
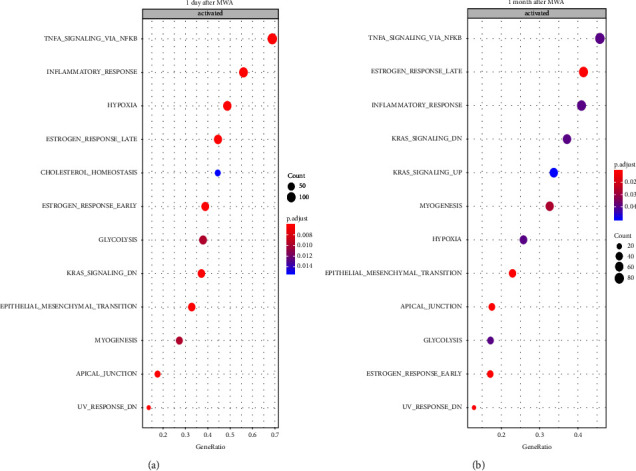
Gene enrichment analysis at 1 day (1 D) and 1 month (1 M) after MWA. GSEA showed hypoxic and glycolytic pathways, and NF-kappa B-mediated TNF*α* downregulation consistent with GO analysis at 1 day (a) and 1 month (b) after MWA. GSEA: gene set enrichment analysis; GO: gene ontology; MWA: microwave ablation.

We identified 12 signal pathways in all the gene enrichment pathways (before, one day after, and one month after MWA) in the malignant group using GSEA. Furthermore, 10 out of these 12 gene enrichment pathways (83.33%) were found to be differentially expressed before treatment, one day after, or one month after treatment. The ten gene-rich information pathways include the NF-kappa B-TNF*α*, inflammatory response, hypoxia, late estrogen response, early estrogen response, glycolysis, epithelial-mesenchymal transition, myocytogenesis, apical node, and UV response downregulation (Figures 4(a) and 4(b)). The GSEA results were consistent with the findings from GO analyses of oxygen transport and oxygen carrier activity one day after MWA, as well as the toll-like receptor signaling pathway one month after MWA.

### 3.7. Cytokine Inflammatory Response

**Figure 5:**
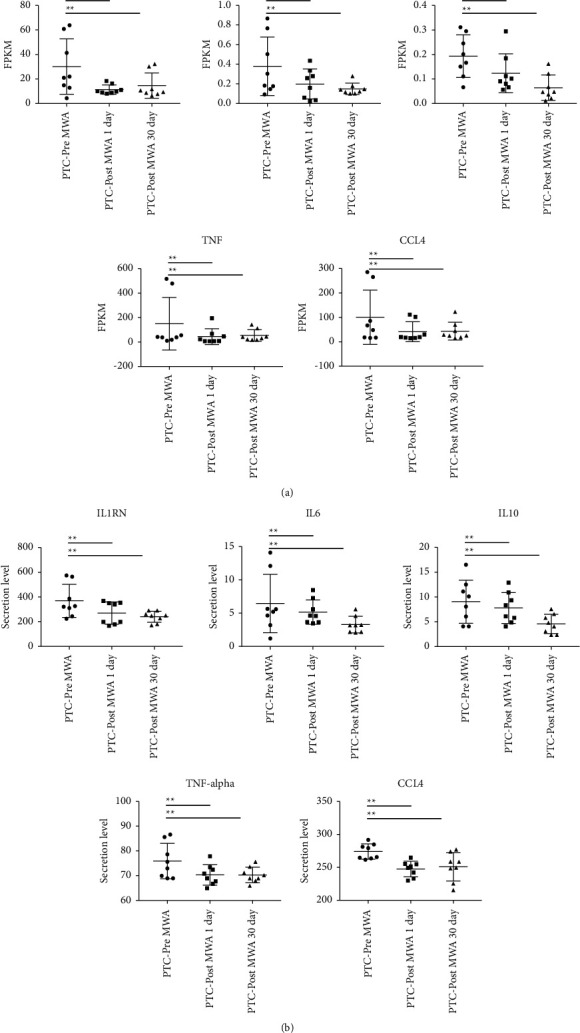
Cytokine inflammatory response after MWA. Downregulation of IL-6, TNF-A, IL-10, and IL-1RN inflammatory regulatory genes (a) validated by the exocytosis expression of proinflammatory cytokines (b). The data are depicted as the mean ± SD, *n* = 8 in each group. ^*∗*^*p* ≤ 0.05; ^*∗∗*^*p* ≤ 0.01; ^*∗∗∗*^*p* ≤ 0.001. MWA: microwave ablation.

The regulatory genes of interleukin-6 (*IL-6*), interleukin-10 (*IL-10*), tumor necrosis factor-A (*TNF-A*), and interleukin-1 receptor antagonist (*IL-1RN*) were downregulated, and the regulatory gene of inflammation-related chemokine ligand *CCL4* was downregulated after MWA (Figure 5(a)). The regulation genes of inflammation-related cytokines and extracellular cytokines changes were validated after MWA, including *IL-6*, *TNF-A*, *IL-10*, and *IL-1RN* (Figure 5(b)).

### 3.8. Tumor-Related Chemokines

**Figure 6:**
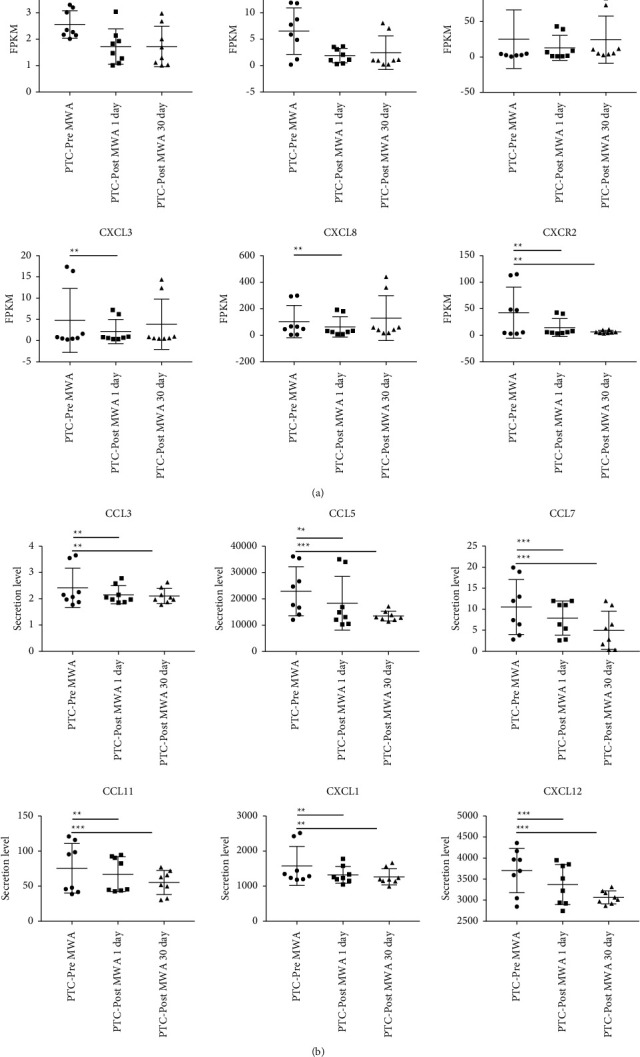
Cytokine antitumor immunity after MWA. Significantly downregulated protumor chemokine-regulated genes (a) validated by exocytosis expression of those chemokines (b). The data are depicted as the mean ± SD, *n* = 8 in each group. ^*∗*^*p* ≤ 0.05; ^*∗∗*^*p* ≤ 0.01; ^*∗∗∗*^*p* ≤ 0.001. MWA: microwave ablation.

The tumor-related chemokines could be downregulated, including inflammatory cytokines and protumor chemokines. After MWA, the expression of protumor chemokine-regulated genes, including *CCR3*, *CXCL1*, *CXCL2*, *CXCL3*, *CXCL8*, and *CXCR2*, was significantly downregulated (Figure 6(a)). As verified by exocytotic chemokines, the expression of tumor-promoting chemokines, including *CCL3*, *CCL5*, *CCL7*, *CCL11*, *CXCL1*, and *CXCL12*, showed a downward trend after MWA (Figure 6(b)).

## 4. Discussion

While immune surveillance is best conducted directly on affected tissues, such as those impacted by inflammatory diseases or tumors in the tumor microenvironment, obtaining these tissues for repeated analysis can be difficult and unethical due to their rarity. Therefore, peripheral blood extraction plays a crucial role. This type of extraction is routinely used in clinics and provides relevant information about human homeostasis that is both accessible and easy to obtain. By taking a few peripheral blood samples during routine clinical follow-up and examination, clinicians can closely evaluate the immune status of each patient, providing valuable information for immune research and clinical applications.

Recent evidence suggests that thermal ablation could change the PBMC balance to enhance systemic antitumor immunity. Zhou et al. [11] found that T cell clones expand with increased T cell receptor diversities for patients with breast cancer after MWA. Activated antigen receptor-mediated signaling pathways are found in B cells. Enhanced interactions between B cells and CD4+ T cells are also found, indicating that B cells are important antigen-presenting cells that initiate CD4+ T cells in the MWA-induced immune response. Wang et al. [12] reported that thermal ablation could increase the frequency and function of CD3^−^CD56^+^ NK cells in the peripheral blood of patients with liver cancer. Takaki et al. [8] demonstrated that tumor ablation could alter the T cell balance by increasing the systemic CTL/T_reg_ ratio.

Our study investigated the effects of MWA on gene expression patterns and found ten differentially expressed pathways before, one day after, and one month after MWA. These pathways were significantly downregulated, including the transcription factor nuclear factor-kappa B (NF-kappa B), inflammatory response, hypoxia, late estrogen response, early estrogen response, glycolysis, epithelial-mesenchymal transition (EMT), and myocytogenesis. These findings suggest that MWA may have a broad impact on gene expression related to inflammation, metabolism, and cellular transformation.

Mesenchymal stromal cells (MSCs) have a crucial role in immune regulation. The immunosuppressive function of MSCs is initiated through the TNF*α*/NF-*κ*B signaling pathway. Increasing evidence suggests that drugs interfering with NF-*κ*B activation can significantly counteract the immunomodulatory effect of MSCs. These findings have important implications for the development of clinical immunosuppression protocols. Specifically, inhibiting NF-*κ*B through i*κ*B kinase *β* or TNF*α* receptor silencing can abolish the immunosuppressive capacity of MSCs [14]. EMT is a critical process that contributes to tumor progression and metastasis, and Twist1 has been identified as a key player in EMT in PTC via the NF-*κ*B pathway [15]. The NF-*κ*B pathway has a crucial role in immune and inflammatory responses by regulating gene expression. It plays a vital role in the development of lymphoid organs, maturation of dendritic cells (DCs), and activation of T cells, all of which are critical components of the immune response.

In the hypoxic microenvironment, hypoxia-inducible factors (*HIFs*) are the primary adaptive mechanism for tumor growth. *HIF* represents the suppression of the antitumor immune response by recruiting tumor-promoting immune cells altering the effector function of immune cells and promoting the production of tumor cytokines, angiogenesis, and ROS production to promote tumor growth [16]. Tumors stimulate inflammation by secreting cytokines, chemokines, and growth factors and promote the recruitment of immune cell population to infiltrate into the tumor microenvironment in a particular range; the inflammatory response can promote tumor growth and metastasis; in addition, EMT can also promote the secretion of inflammatory cytokines; EMT is related to the inflammatory response and tumor progression [17]. Therefore, the antitumor immune response is closely related to the downregulation of the NF-kappa B-TNF*α* pathway, hypoxia, glycolysis, inflammation, and EMT. Estrogen regulation is critical for thyroid cancer outcomes (angiogenesis, and metastasis) [18]. Estrogen can also play an influential regulatory role at the level of innate and adaptive immune systems [19], and it has been reported that estrogen may affect T cell development and alter the ability of telomerase to participate in the T cell receptor response [20]. Downregulation of the early and late estrogen response can achieve the antitumor response to a certain extent and can weakly change innate and adaptive immunity levels.

By differential gene screening, we found that a total of 10 genes changed before and after one day and one month, including *AC118658.1*, *BCL3*, *CELF2-AS1*, *MXD1*, *MYH10*, *NR6A1*, *PDGFB*, *PELI1*, *PFKFB3*, and *TREML4*; except *PDGFB*, the other nine differential genes were downregulated after treatment.

*BCL3*, *MYH10*, NR6A1, and *PFKFB3* are all protumor genes, which are all downregulated after MWA treatment. Proto-oncogene *BCL3* can induce the survival and proliferation of cancer cells. Previous studies have found that *BCL3* is highly expressed in ovarian cancer tissues. In addition, interferon (*IFN*) can promote the expression of *BCl3* in ovarian cancer cells and increase the transcription level of the immune checkpoint molecule PD-L1 [21]. Silencing the *MYH10* decreased *MTA-1*, *MP-2*, *MMP-9*, and vimentin while increasing expression of *TIMP-2*, E-cadherin, and collagen one at both protein and mRNA levels and inhibited the Wnt/b-catenin pathway. In human glioma cell lines, silencing the *MYH10* can inhibit the Wnt/b-catenin pathway to reduce tumor cell migration and invasion, which may regulate the human glioma tumor microenvironment and inhibit epithelial-mesenchymal transition [22]. Studies have shown that the expression level of *NR6A1* in cells is closely related to the disease progression of prostate cancer. The increased *NR6A1* expression was significantly associated with the Gleason score, advanced pT stage, and cancer cell proliferation [23]. The high expression of endogenous *PFKFB3* promotes glycolysis, and the blockade of *PFKFB3* reduces angiogenesis in vivo and in vitro. Therefore, reducing the *PFKFB3* expression targets lowering glycolysis and related pathological angiogenesis [24, 25]. We hypothesized that the inhibition of *PFKFB3* could reduce the inflammatory response in tumors and then inhibit tumor growth [26]. *PELI1* is a gene associated with the negative regulation of T cells, which is downregulated after MWA. Chang et al. showed that *PELI1* is a critical regulatory gene for T cell activation and autoimmunity, and downregulation of *PELI1* can positively regulate and activate T cells [27, 28]. Therefore, the present study found that the downregulation of oncogenic genes such as *BCL3*, *MYH10*, *NR6A1*, and *PFKFB3* after MWA indicates the effectiveness of the MWA and antitumor immune response.

One day after MWA of malignant thyroid nodules, GO enrichment analysis of differential genes showed that the enriched pathways mainly included the oxygen transport process, oxygen carrier activity process, hydrogen peroxide catabolism process, gas transport process, and bicarbonate transport process. According to the mRNA sequencing results, hypoxia can be relieved by the PBMC level to achieve the antitumor immune effect after MWA, consistent with the GSEA results. One month after MWA, GO enrichment analysis showed that the enrichment pathway was mainly the positive regulation of the toll-like receptor signaling pathway. Innate immunity is the first line of defense against pathogen invasion. Toll-like receptors (TLRs) are the primary sensors to detect microbial components and trigger innate immune responses. All TLR signaling pathways ultimately activate NF-kappa B. The latter controls the expression of a range of inflammatory cytokine genes [29]. Thus, positive regulation of toll-like receptor signaling is closely related to antitumor immunity induced by enhanced antigen presentation by DCs after MWA.

About 20% of all tumors are estimated to arise from chronic inflammation, and chronic thyroiditis can cause thyroid cancer [30]. Exocytotic cytokines associated with systemic proinflammatory responses, such as IL6, IL-10, TNF-A, IL-1RN, and chemokine CCL4, were downregulated as were corresponding mRNA level regulatory genes, consistent with the GSEA results, namely, the inflammatory response pathway after treatment. The downregulation of NF-kappa B signaling also inhibits proinflammatory cytokine secretion after MWA [31]. In our study, the downregulation of the NF-kappa B-TNF*α* pathway was consistent with the downregulation of CXCL8 regulatory genes in PBMC and exocytotic TNF-A. The inflammatory response pathway was also downregulated in GSEA after MWA, indicating that MWA induced an indirect antitumor immune effect.

Chemokines are a group of small proteins that elicit normal physiological and immune responses primarily by recruiting specific cell populations to infected tissues or sites of malignancy. Like other tumor cells, thyroid cancer cells can influence thyroid cancer progression in many ways by disrupting the chemokine system and altering the ability of cells to migrate into the tumor microenvironment [32]. According to transcriptome sequencing, antitumor chemokine regulatory genes (CXCR3) were upregulated, and protumor chemokine regulatory genes (CCR3, CXCL1, CXCL2, CXCL3, CXCL8, and CXCR2) were downregulated after MWA in thyroid cancer. At the same time, extracellular tumor-promoting chemokines, such as CCL3, CCL5, CCL7, CCL11, CXCL1, and CXCL12, were also downregulated. Therefore, the change in the chemokine level also indicated the antitumor immune effect after MWA.

Our study has several limitations. First, the number of cases is relatively small, the number of transcriptome sequencing samples is small, and the follow-up time for recurrence and metastasis is relatively short. Second, transcriptome sequencing did not quantify specific cell numbers before and after MWA. That will be the goal of our further research. Third, in clinical practice, achieving repeated detection of thyroid tumor tissue before and after MWA is challenging and fails to meet ethical requirements. Instead, we opted for repeated measurements of peripheral venous blood in PTMC patients before and after MWA to capture systemic immune changes, which could offer valuable insights into antitumor immunity.

## 5. Conclusions

In this study, we present a comprehensive overview of the systemic immune response triggered by thermal ablation for papillary thyroid microcarcinoma. Importantly, our findings provide a foundation for identifying possible approaches to enhance systemic antitumor immunity in thyroid cancer via microwave ablation. In the future, peripheral venous blood is expected to reflect the effectiveness of ablation for thyroid microcarcinoma.

## Figures and Tables

**Table 1:** The primer sequences for the qRT-PCR.

Gene name	Forward primer	Reverse primer
*BCL3*	AACCTGCCTACACCCCTATAC	CACCACAGCAATATGGAGAGG
*PFKFB3*	ATTGCGGTTTTCGATGCCAC	GCCACAACTGTAGGGTCGT
*NR6A*	GGGATGAACCGGAAGGCTATC	GGCTGGTTGCTCTCCGAAG

RT-PCR: reverse transcription-polymerase chain reaction.

**Table 2:** Patient characteristics and detailed ablation parameters.

No	Age	Gender	Height (cm)/weight (kg)	Nodule maximum size (cm)	Voltage (W, MHZ)/time (s)
1	34	Female	166/46	0.5	30 W, 2450 MHZ, 53 s
2	33	Male	162/75	0.55	30 W, 2450 MHZ, 1 min 10 s
3	38	Female	155/50.5	0.4	30 W, 2450 MHZ, 1 min 2 s
4	44	Male	175/90	0.3	30 W, 2450 MHZ, 48 s
5	36	Female	160/64	0.4	30 W, 2450 MHZ, 2 min 21 s
6	51	Male	168/75	0.3	30 W, 2450 MHZ, 36 s
7	55	Male	170/70	0.6	30 W, 2450 MHZ, 34 s
8	36	Female	156/46	0.2	30 W, 2450 MHZ, 21 s

**Table 3:** Differential genetic expression after MWA.

Group	Total genes	DEGs	Enrolled DEGs	Upregulated gene/LogFC >0	Downregulated gene/LogFC <0
1 day after MWA	36686	277	91	27	64
1 month after MWA	36576	235	37	6	31

FC: fold change; DEGs: differential expression genes; MWA: microwave ablation.

**Table 4:** Analysis of cochanging genes before, 1 day, and 1 month after MWA.

Gene name	Type	Fold change	*p* value	Up/down	Statistical difference	Before MWA	One day after MWA	One month after MWA	Function
*AC118658.1*	Sense intronic	3.098825	0.0021793	Down	Yes	2.231332	0.580330	0.722498	—
*BCL3*	Encoding protein	1.702173	0.0038863	Down	Yes	29.44939	19.36760	17.12488	Promote the tumor
*CELF2-AS1*	Antisense	1.672665	0.0336624	Down	No	10.43871	7.195725	6.447062	—
*MXD1*	Encoding protein	2.134729	0.0014669	Down	Yes	43.11530	23.30597	21.00734	—
*MYH10*	Encoding protein	1.808122	0.0002892	Down	No	1.944659	1.290079	1.057608	Promote the tumor
*NR6A1*	Encoding protein	2.176488	0.0011333	Down	Yes	1.154163	0.560143	0.506250	Promote the tumor
*PDGFB*	Encoding protein	0.536241	0.0007647	Up	No	1.565460	2.951772	2.844862	—
*PELI1*	Encoding protein	1.838839	0.0008184	Down	No	49.15218	29.67204	28.67416	Negative regulation of T cell
*PFKFB3*	Encoding protein	2.081658	0.0002157	Down	Yes	24.45271	11.87692	11.40871	Promote the tumor
*TREML4*	Encoding protein	2.621220	0.0087561	Down	Yes	1.167181	0.521761	0.481796	Antigen-presenting

MWA: microwave ablation.

## Data Availability

The data that support the findings of this study are available from the corresponding author.
